# Photoperiodic Modulation of Circadian Clock and Reproductive Axis Gene Expression in the Pre-Pubertal European Sea Bass Brain

**DOI:** 10.1371/journal.pone.0144158

**Published:** 2015-12-07

**Authors:** Rute S. T. Martins, Ana Gomez, Silvia Zanuy, Manuel Carrillo, Adelino V. M. Canário

**Affiliations:** 1 Comparative Endocrinology and Integrative Biology group, Centre of Marine Sciences (CCMAR), University of Algarve, Campus de Gambelas, Faro, Portugal; 2 Department of Fish Physiology and Biotechnology, Instituto de Acuicultura de Torre la Sal, Consejo Superior de Investigaciones Científicas (CSIC), Torre la Sal, Castellón, Spain; McGill University, CANADA

## Abstract

The acquisition of reproductive competence requires the activation of the brain-pituitary-gonad (BPG) axis, which in most vertebrates, including fishes, is initiated by changes in photoperiod. In the European sea bass long-term exposure to continuous light (LL) alters the rhythm of reproductive hormones, delays spermatogenesis and reduces the incidence of precocious males. In contrast, an early shift from long to short photoperiod (AP) accelerates spermatogenesis. However, how photoperiod affects key genes in the brain to trigger the onset of puberty is still largely unknown. Here, we investigated if the integration of the light stimulus by clock proteins is sufficient to activate key genes that trigger the BPG axis in the European sea bass. We found that the clock genes *clock*, *npas2*, *bmal1* and the BPG genes *gnrh*, *kiss* and *kissr* share conserved transcription factor frameworks in their promoters, suggesting co-regulation. Other gene promoters of the BGP axis were also predicted to be co-regulated by the same frameworks. Co-regulation was confirmed through gene expression analysis of brains from males exposed to LL or AP photoperiod compared to natural conditions: LL fish had suppressed *gnrh1*, *kiss2*, *galr1b* and *esr1*, while AP fish had stimulated *npas2*, *gnrh1*, *gnrh2*, *kiss2*, *kiss1rb* and *galr1b* compared to NP. It is concluded that fish exposed to different photoperiods present significant expression differences in some clock and reproductive axis related genes well before the first detectable endocrine and morphological responses of the BPG axis.

## Introduction

Puberty is a process by which a juvenile animal acquires reproductive competence [1]. During this process major hormonal, physical and behavioural changes occur. In mammals, puberty initiates in the brain and requires the activation of cellular mechanisms that control and regulate different levels of the brain-pituitary-gonadal axis (BPG). An increase in hypothalamic kisspeptin (KISS1) stimulates the production of gonadotropin-releasing hormone (GNRH) and pituitary gonadotropins, luteinizing hormone (LH) and follicle stimulating hormone (FSH), which stimulate gonadal maturation and steroid production [2]. Several internal and external factors including light perception strongly influence the onset of puberty [3].

Light variations are integrated at the molecular level in the suprachiasmatic nucleus (SCN) by complex feedback loops of the core clock genes, e.g. circadian locomotor output cycles kaput (*CLOCK*), neuronal PAS domain-containing protein 2 (*NPAS2*), period (*PER 1–3*) [4]. As key regulators of seasonal timed processes, clock gene mutations have been associated with reproductive traits in *Drosophila* [5], humans [6] and mice [7] and [8]. In mammals, SCN outputs towards GNRH and KISS1 neurons [9,10] have been shown to regulate the synchronization of ovulation and induction of the LH surge [11] suggesting a direct effect of the SCN on the reproductive axis. However, recent work has shown that the regulation of GNRH/KISS1 neurons is far more complex than a simple hierarchical regulation of these neurons by the SCN circadian clock genes as desynchronization of the dorsomedial (dm) nuclei of the SCN affects KISS1 neurons in the hypothalamus in a light-dark cycle independent manner [11]. Additional work in mammalian neuronal cell lines further highlighted that the GNRH expressing cells also express locally circadian clock genes that, when deleted [12] or overexpressed [13] result in a reduction or an increase in GNRH pulses. These studies clearly demonstrate that reproductive function and rhythmicity involves multiple clock oscillators that are not exclusively located in the central SCN.

In male European sea bass, *Dicentrarchus labrax*, (henceforth sea bass) puberty normally occurs during the second year of life but in aquaculture a high proportion (up to 30%) undergo precocious maturation within their first year [14]. In this species reproduction is a seasonal event and specific light regimes can be used to inhibit, delay or advance the onset of puberty and first spermiation [15,16]. Continuous light (LL) regimes delay gametogenesis progression and inhibit the onset of puberty thereby suppressing the incidence of precocious males [17–19]; expanded, compressed, or a shift from a long to a short photoperiod (SL) in spring advance spermiation [14,19,20]. Recently, it has been shown that there is a window of highest sensitivity to photoperiod treatments and, at least for the LL regime, a two month exposure to continuous light during September, when gametogenesis starts, is an effective inhibitor of precocious maturation in sea bass [21]. Nonetheless, how the different photoperiod regimes exert their influence on reproductive processes is still largely unknown.

In sea bass as gonadal development proceeds towards puberty, pituitary levels of gonadotropin-releasing hormone 1 (Gnrh1) are higher than those of Gnrh2 and Gnrh3, plasma Lh levels start to increase but androgen levels remain low [16]. Thus, although pituitary Gnrh1 and Lh are elevated prior to puberty, not enough androgen is produced by the testes to promote germ cell maturation. As spermatogenesis progresses, the levels of plasma testosterone (T), 11-ketotestosterone (11-KT) [16], pituitary *lh* and *fsh* β-subunit mRNA increase and the progression towards germ cell maturation and sperm production occurs [18]. This suggests that a negative feedback signal, possibly originated from the gonads, and/or a critical trigger of the BPG axis, is activated at the onset of puberty.

Kisspeptin has been proposed as a likely candidate for the activation of the BPG axis during the onset of puberty. In teleost fish, two paralog genes (*kiss1* and *kiss2*) encode kisspeptins and up to four different genes encode kiss receptors (*kissr*) [22]. In common with what has been observed in mammals, injections of kisspeptin (kiss2) in zebrafish, *Danio rerio* [23] and sea bass [24], increase gonadotropin release [25]. In fishes, experimental evidence of crosstalk between the circadian clock and the activation/regulation of KISS-GNRH systems is still lacking, and to date the mechanisms involved in triggering pubertal onset are still unknown.

In the present study we aimed to identify genes that are involved in the early responses to either puberty accelerating (AP) or inhibiting (LL) photoperiods in the brain of pre-pubertal sea bass and to clarify whether some components of the circadian clock and reproduction-related genes are part of this response. We hypothesized that if the activation of puberty requires the orchestrated regulation of circadian clock genes (*clock*, *npas2* and *arntl*) for the perception of the light stimulus, and reproduction related genes *(gnrh2*, *kiss1*, *kiss2* and *kissrs)* for BGP axis activation the two sets of genes are likely to share the same regulatory elements, similarly spatially organized within their promoters in frameworks (i.e. conserved groups of transcription factors appearing in the same order and equally spaced) [26,27]. Interestingly, not only we found common promoter frameworks in the two groups of genes, we have also identified an additional 173 vertebrate gene promoters that share the same frameworks. We analysed the response of some of the identified genes and candidate network partners to different photoperiod conditions in sea bass predicted to enter or not puberty, and found specific patterns of co-regulated gene expression for LL and AP photoperiods. We show that photoperiod regimes affect the brain mRNA expression of circadian and reproductive axis related genes prior to the activation of the endocrine BPG axis, suggesting that puberty onset requires coordination and possibly co-regulation of clock and BGP axis genes.

## Material and Methods

### Identification of promoter frameworks

The 1.5 Kb putative promoter sequences of the genes analysed in this study were retrieved from the zebrafish zv9 genome assembly at Ensembl database (www.ensembl.org)—*kiss1* (Chr.11: 24248322–24249833), *kiss2* (Chr.4:15894109–15895771, *kiss1rb* (Chr.2: 33512576–33514087), *kiss2rb* (Chr.5: 72415097–72416608), *arntl1a* (Chr.25: 18397999–18399503), *arntl1b* (Chr.7:67978425–67979898), *clock* (Chr. 20: 22200972–22202477), *clock3* (Chr.1: 18174667–18176172), *npas2* (Chr.5: 24573559–14575064) and *gnrh2* (Chr.21: 14027250–14028860)—and from the European sea bass genome assembly (dicLab v1.0c at http://seabass.mpipz.de)—*kiss1* (LG1A:1602662–1606367), *kiss2* (LGx:1084174–1087770), *kiss1rb* (LG10:16049229–16063479), *kiss2rb* (LG20:15036333–15040834), *arntl1a* (LG5:15033014–15038159), *arntl1b* (LG6:23295633–23321243), *clock* (LG7:16250759–16266546), *clock3* (LG4:20865175–20889027), *npas2* (LG14:11922193–11945535) and *gnrh2* (LG1A:10673952–10675997).

Promoter sequences were compared by definition of a similar pattern or framework of TFBSs (software Frameworker, Genomatix, www.genomatix.de) following a previously described methodology [27–29]. A framework is defined as a set of two or more TFBSs with a specific order, strand orientation, and distance range between the individual TFBSs. Position weight matrices were used to represent the TFBSs using default parameters. The matrix family library Version 7.0 (Genomatix) was used for the analysis. To define specificity and selectivity of the promoter sequences, the existence of complex models composed of three or more common TFBSs conserved in order, space (5–500 bp, with 10–20 bp variance between promoters) and orientation was analysed in orthologous promoters. Each of the complex models identified was then compared to a background promoter sequence set of 5000 human promoters with a p-value assigned to each framework. Only models with a p-value <1E^-10^ were selected for analysis. The respective patterns of common TFBSs were tested on promoter libraries compiled from primate, rodent, other mammalian and other vertebrate species (Genebank release 162) using the program Model Inspector from the Gems Launcher Suite (Genomatix).

The zebrafish *clock/npas2/arntl-kiss/kissr- gnrh2* co-regulated genes identified by model inspector were annotated using DAVID bioinformatics resources (version 6.7). Genes were annotated using the Gene Ontology database, clustered by biological function and an enrichment score assigned to each cluster (EASE score p < 0.05). A False Discovery Rate test was applied to the clusters and only biological function clusters with a q-value <0.05 were considered. Pathway analysis software was used to draw the conserved networks (Ingenuity systems, IPA version 7.6). The same analysis performed without the *gnrh2* promoter sequence was used to evaluate the specificity of the identified promoter frameworks and model inspector results.

### Photoperiod experiments

The sea bass brain samples analysed herein were obtained as part of a larger study. Five month old sea bass were purchased from a commercial hatchery February 12th, 2008 (Gravelines, France) and maintained at the CSIC fish facilities (Torre de La Sal, Castellón, Spain) under natural photoperiod.

**Fig 1:**
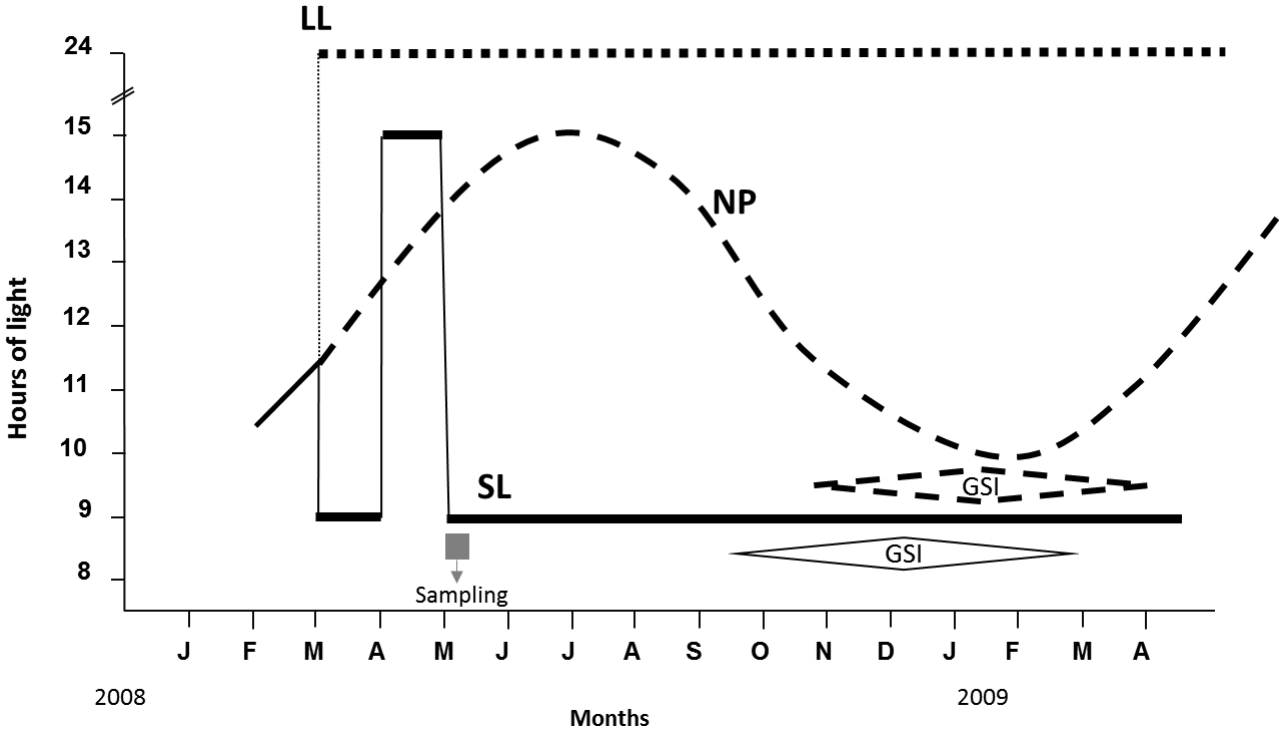
Experimental setup and photoperiod regimes used in this study. Sea bass were purchased and maintained from February until March under natural photoperiod (black line). On the 9^th^ of March the fish were separated into three groups, one exposed to simulated natural photoperiod (NP, broken line), a second to continuous light (LL, spotted line) and a third to 9L: 15D (AP, continuous line). On the 2^nd^ of April the AP group was switched to a long day light regime (15L: 9D) and on the 4^th^ of May a short day regime (9L: 15D). Fish were sampled on the 5^th^, 6^th^ and 8^th^ of May. Horizontal diamonds indicate the spermiation period for the AP and NP groups. The LL group did not spermiate.

On March 9^th^, 2008, three groups of fish in triplicate were exposed to different light regimes (Fig 1). The NP group had lights turned on and off sharply with timings adjusted daily according to the natural light: dark regime, the LL group was maintained under continuous light (24L:0D), and the AP group under a combination of three constant photoperiods in a sequentially manner: a) From March 9^th^ until the 1^st^ of April, fish were exposed to a constant short photoperiod (9h light: 15 h dark, corresponding the "lights on" at 8:30 am and the "lights off" at 17:30 pm, respectively, to the "sun rise" and the "sun set" at the time of the winter solstice); b) between the 2^nd^ of April and the 3^rd^ May, fish were exposed to a constant long photoperiod (15h light: 9h dark, corresponding the "lights on" at 6:30 am and the "lights off" at 21: 30 pm, respectively, to the "sun rise" and the "sun set" at the time of summer solstice) and c) on the 4^th^ of May, the fish were switched back to constant short photoperiod as above until the end of the experiment.

On the 2^nd^ of April fish from all experimental groups were size graded into two subgroups: fast growing fish (large ≥ 6g) and slow growing fish (small ≤3g) (S1 Fig), in order to separate fish that were expected to reproduce in the first spawning season (larger fish; precocious) from those that would not reproduce (small). Fish from all experimental groups were sampled on the 5^th^, 6^th^ and 8^th^ of May starting at 9:00 a.m. and always in the same order, LL followed by AP and NP (Fig 1).

Briefly, the fish were sacrificed and the whole brain was removed and stored in RNA later until analysis. The present study only reports the analysis of larger fish. This experiment was conducted in accordance with Spanish (Royal Decree 53/2013) and European (2010/63EU) legislation concerning the protection of animals used for experimentation. The experimental manipulations and housing of the animals was approved by the Welfare Committee of the Instituto de Acuicultura de Torre de la Sal (IATS) (Register Number 09–0201), under the supervision of the Ministry of Rural and Marine Environment. The behaviour and health of all animals was monitored visually each day and no evidence of infection, modified behaviour or mortality was observed during the experiments. All fish were sacrificed after administration of a lethal dose of 2-phenoxyethanol, and all efforts were made to minimize suffering.

### RNA isolation and cDNA synthesis

Total RNA was extracted using an automated system, Maxwell 16 Mdx (Promega) and following the manufacturer’s indications. The integrity and purity of the total RNA isolated was assessed using 1% agarose gel electrophoresis and quantified in a NanoDrop 1000 Spectrophotometer (Thermo Fisher Scientific, USA). Total RNA was treated with DNase (DNA-free kit, Ambion, UK) and cDNA synthesis carried out in 20 μl reactions containing 500 ng of DNase-treated RNA, 200 ng of random hexamers (Jena Biosciences, Germany), 100 U of RevertAid (Fermentas, Thermo Fisher Scientific, USA) reverse transcriptase and 8 U of RiboLock RNase Inhibitor (Fermentas). Reactions were incubated for 10 min at 25°C and 60 min at 42°C, followed by enzyme inactivation for 10 min at 70°C, and storage at -20°C until use in the amplification of sea bass orthologue genes and RT-qPCR.

### Isolation of gene targets

Sets of primers were designed to amplify cDNA fragments from sea bass *clock1* and *npas2* (primer combinations Fw1/Rv2, Table 1). The primers used to amplify the probes for *kiss2*, *kiss1rb* and *esr1* were the same as those used for quantitative real time PCR (qPCR) (Table 1). cDNAs probes for sea bass *gnrh1* and *gnrh2*, galanin receptor 1a (*galr1a*) and galanin receptor 1b (*galr1b*) were generated as previously reported [30]. Briefly, each gene was amplified from cDNA by reverse transcription-polymerase chain reaction (RT-PCR) in 25 μl containing 1 μl of cDNA (from whole larvae, or brain or liver of adult sea bass), 10 pmol each primer, 40 μM dNTPs and 0.5 U DreamTaq DNA Polymerase (Fermentas), in 1x DreamTaq buffer. Cycling conditions were 5 min at 95°C, 35 cycles of 20 sec at 95°C, 20 sec at 58–62°C depending on the primer pair and 1 min at 72°C, followed by 5 min at 72°C. Amplified targets were gel-purified, inserted into pGEM-T Easy vector (Promega, Southampton, UK) and their identity confirmed by sequencing.

**Table 1:** List of primers used in RT-qPCR and accession numbers of corresponding genes.

Gene name	Fw/Rv^a^	Primer sequence (5'-3')	Accession number
*galr1a*	Fw1	GCTGCCACTGCCTGGCG	KF878115
	Rv1	CAAACGTTGGTGCAGTTAGTC	
*galr1b*	Fw1	CTGGTGCCGGTTGCCCAGC	KF878116
	Rv1	CAAAGCATTTTACTGGTCTGAG	
*clock1*	Fw1	GCAGTCATGGTCCCCACAAC	DLAgn_00181130
	Rv1	GGCTGCTGGGCAATGCTGA	
	Rv2	GGAACTGCGTTTGCTGCTG	
*npas2*	Fw1	AGTCAGATATGGTGGACC	DLAgn_00040750
	Rv1	GAAGTCAGCAGCAATGGGG	
	Rv2	CAGACATCGATCATATCG	
*kiss1rb*	Fw1	CGTGACAGTCTACCCCCTGAA	JN202446
	Rv1	TCCAAATGCAAATGCTGACAA	
*kiss2*	Fw1	ACTCCTGCGGTCGTTGCACAGG	FJ008915
	Rv1	ATGAGGCTCGTGGCTCTGGTCGT	
*esr1*	Fw1	TGGGCGATGGATGCGGTGAGC	AJ505009
	Rv1	CCTGCAGAGCTGTGCAGCGG	
*gnrh1*	Fw1	ACAAACCTTCGCACTGCGGCTG	AF224279
	Rv1	CGTCGCCACGTGTGGGAAGCTC	
*gnrh2*	Fw1	CCTGGTGGGACGTTGGGACACC	AF224281
	Rv1	TCCTCCGAAATCTCTGAAGTGC	
*actb*	Fw1	TGACCTCACAGACTACCT	DLAgn_00070150
	Rv1	GCTCGTAACTCTTCTCCA	
*eef1a*	Fw1	GACACAGAGACTTCATCAAG	DLAgn_00176010
	Rv1	GTCCGTTCTTAGAGATACCA	

^a^ Fw- forward primer, Rv-reverse primer

### Quantitative real time PCR analysis

qPCR was performed in duplicate reactions (with less than 5% variation between replicates) in 96-well micro plates (Bio-Rad) using an ICycler iQ^™^ Real-Time PCR Detection System (Bio-Rad). Reactions were set-up using 2 μl of diluted cDNA (1:10), 7.5 μl of SsoFast EvaGreen supermix (Bio-Rad) and 300nM of each primer (primer combinations Fw1/Rv1, Table 1). All qPCR primers used in the study had an efficiency of 95% or greater. The PCR cycling conditions were optimized for the iCycler as suggested by the manufacturer. Briefly, samples were denatured at 95°C for 30 sec, followed by 45 cycles of 95°C for 5 sec for denaturing, 58–62°C for 10 sec for annealing. Standards were prepared by diluting plasmid clones of target genes. As reference genes we used the beta actin (*actb*) and elongation factor 1 (*eef1a*) as we found no significant differences (p<0.05) in their expression between cDNA samples within and between the experimental treatments (tested by one-way analysis-ANOVA). Thus, the absolute quantities of each target gene were normalized against the geometric mean of the level of expression of the reference genes to remove differences in reverse transcription efficiencies. Negative controls (cDNA synthesis reaction containing the mRNA template but no reverse transcriptase) were included in all runs to confirm the absence of genomic DNA contamination. Statistical analysis of the qPCR results between photoperiod groups was performed using two-way analysis of variance (ANOVA) on untransformed or in log-transformed data when the assumptions of ANOVA were not met. The level of significance was taken at 5%.

## Results

### Identification of co-regulated gene networks

Of the zebrafish and sea bass clock gene promoters analysed, *clock1*, *npas2*, *arntl1a* and *arntl1b* share 10 common frameworks of 3 TFBS in the same order and spacing with the reproduction genes *kiss1*, *kiss2*, *kiss1rb*, *kiss2rb* and *gnrh2*. The *clock3* promoter did not contain all of the identified frameworks and was removed from the analysis. The identified promoter frameworks were composed of different combinations of the following transcription factor binding sites: V$HOXF, V$BRNF, V$CART, V$FKHD, V$CREB, V$SORY, V$HOMF and V$OCT1 (S1 and S2 Tables).

**Fig 2:**
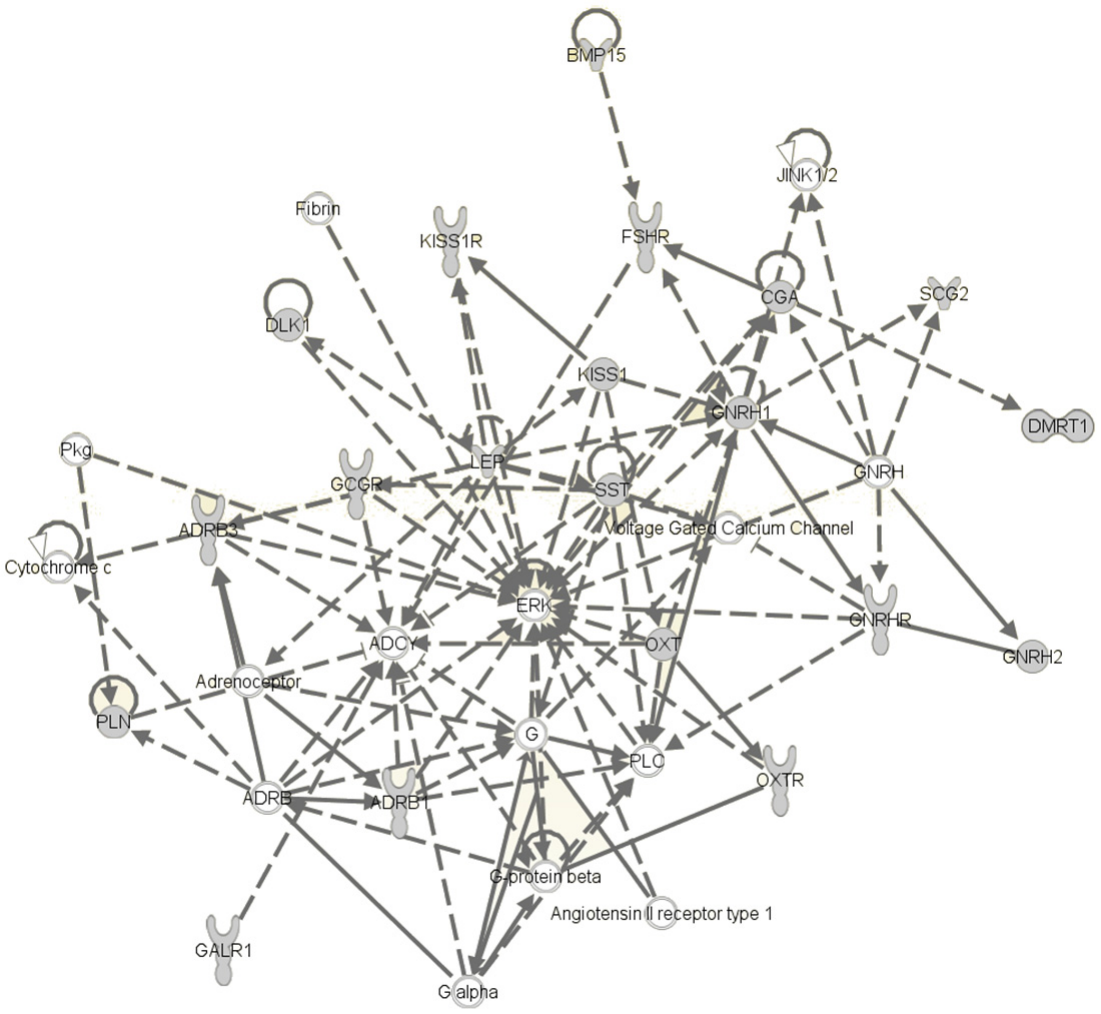
Reproduction related gene network predicted by promoter framework analysis. The genes presented in this network were predicted to be co-regulated with the circadian clock-*gnrh2*-*kiss*/*kissr* genes. Solid lines indicate direct interactions between network partners (nodes), broken lines indicate indirect actions between nodes. A black arrow with a solid line indicates that one node acts on the other and a black arrow with a broken line indicates the node acts indirectly on another node, while a white arrow with a solid line indicates interaction between nodes involving translocation.

The same frameworks were present in the other 173 vertebrate gene promoters suggesting co-regulation by the same transcription factors as *clock*/*npas2*/*arntl1*-*kiss*/*kissr*-*gnrh2* (S3 Table). When *gnrh2* was removed from the analysis a new set of 157 gene promoters were identified that shared the same frameworks as *clock*/*npas2*/*arntl1*-*kiss*/*kissr* (S4 Table). However, only 5 genes were common between the two sets of gene promoters indicating the specificity of co-regulation between the sets of promoters when *gnrh2* is included or not. In the list which included *gnrh2* the biological process categories most significantly over- represented were (1) response to hormone levels, (2) negative regulation of apoptosis, (3) regulation of hormone levels, (4) multicellular organisms homeostasis and (5) gland development (S5 Table). In the list without *gnrh2* the biological process categories most significantly over- represented were (1) vasculature development and angiogenesis, (2) regulation of cell motion/migration, (3) epithelium and gland development, (4) regulation of cell death and (5) regulation of protein kinase cascade (S6 Table). The exclusive over-representation of hormone related categories in the *gnrh2* specific analysis led us to focus only on the corresponding annotation cluster (Fig 2, S5 Table).

### Response of network partners to different photoperiod regimes

The predicted co-regulation of *clock*/*npas2*/*arntl1*- *kiss*/*kissr*-*gnrh2* and network partners and response to light conditions was analysed in brains from sea bass juveniles subject to long to short (AP) and continuous (LL) photoperiods compared to natural photoperiod (NP, control). The proportion of fish that achieved maturity (stage V, spermiating) in December was 17% for NP, 16% for AP and 0% for LL.

**Fig 3:**
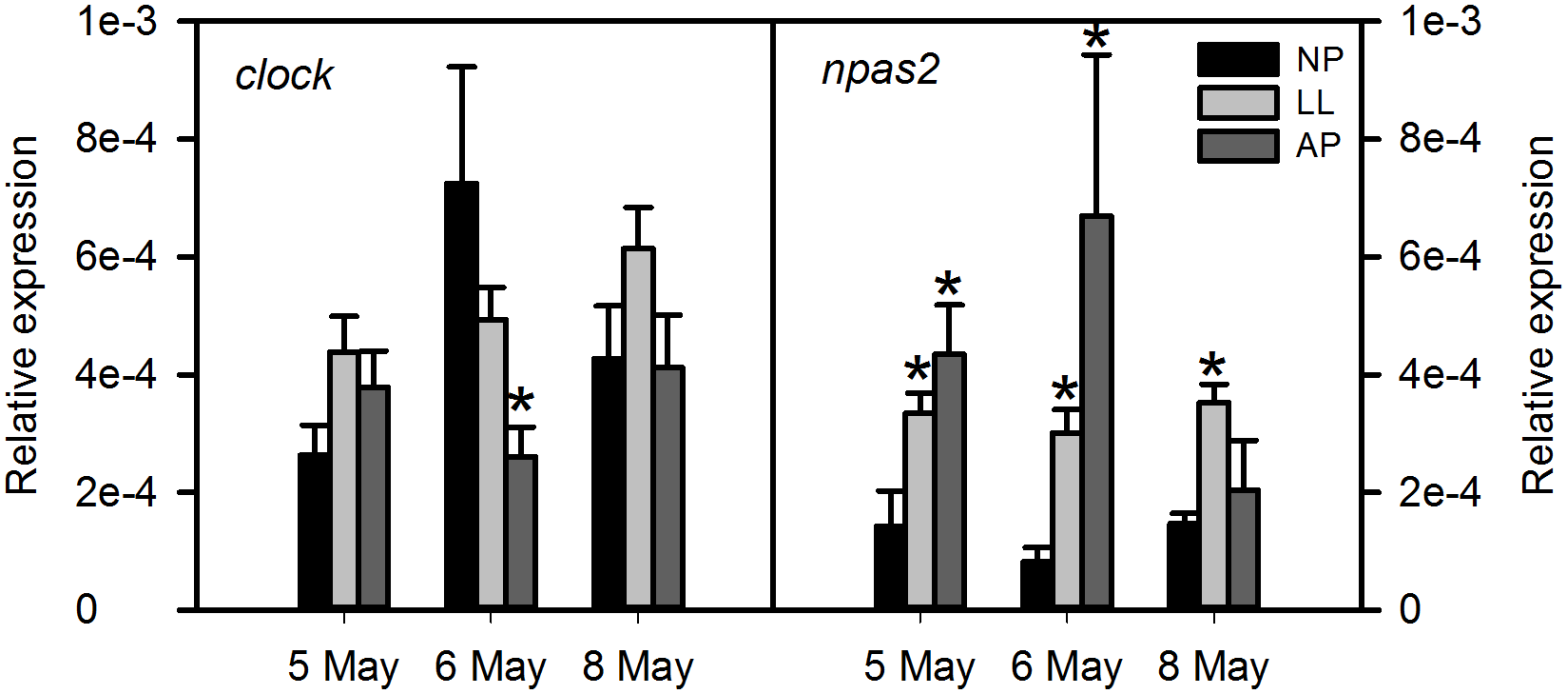
Transcript levels of clock genes in the brain of sea bass exposed to different photoperiods. qPCR analysis was used to measure the transcript levels of *clock1* and *npas2* genes in whole brains of fish exposed to simulated normal (NP), accelerating (AP) and continuous light (LL). Each value is the mean ± SEM (n = 6 fish per group at each sampling time) of the relative expression. Asterisk (*) indicates significant differences of AP and LL fish relative to control fish (NP) (P<0.05).

**Fig 4:**
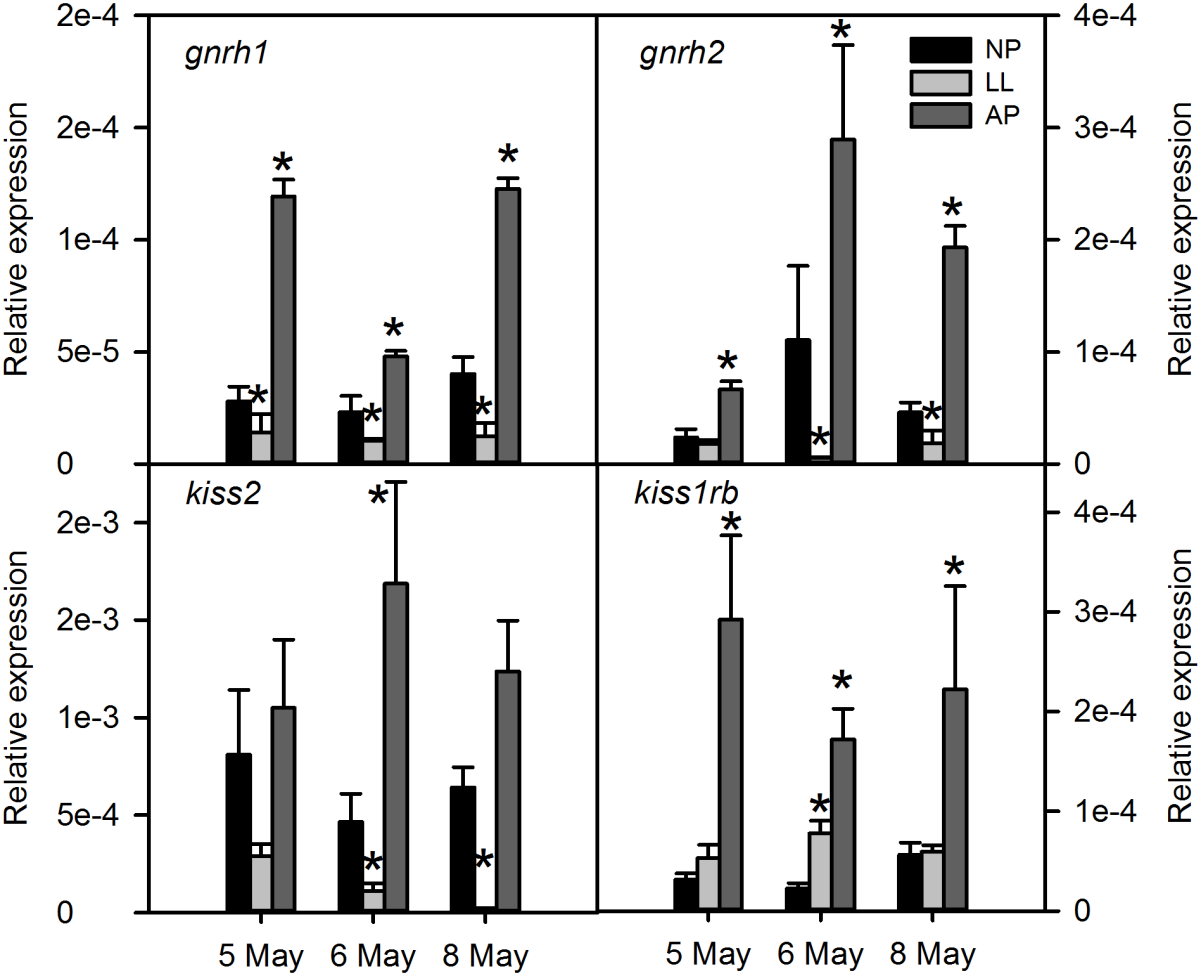
Transcript levels of reproductive axis genes in the brain of sea bass exposed to different photoperiods. The transcript levels of the predicted reproductive axis-related genes (*gnrh1*, *gnrh2*, *kiss2* and *kiss1rb*) was measured by qPCR in whole brains of sea bass exposed to simulated normal (NP), accelerating (AP) and continuous light (LL). Each value is the mean ± SEM (n = 6 individual fish per group at each sampling time) of the relative expression. Asterisk (*) indicates significant differences of AP and LL fish relative to control fish (NP) (P<0.05).

**Fig 5:**
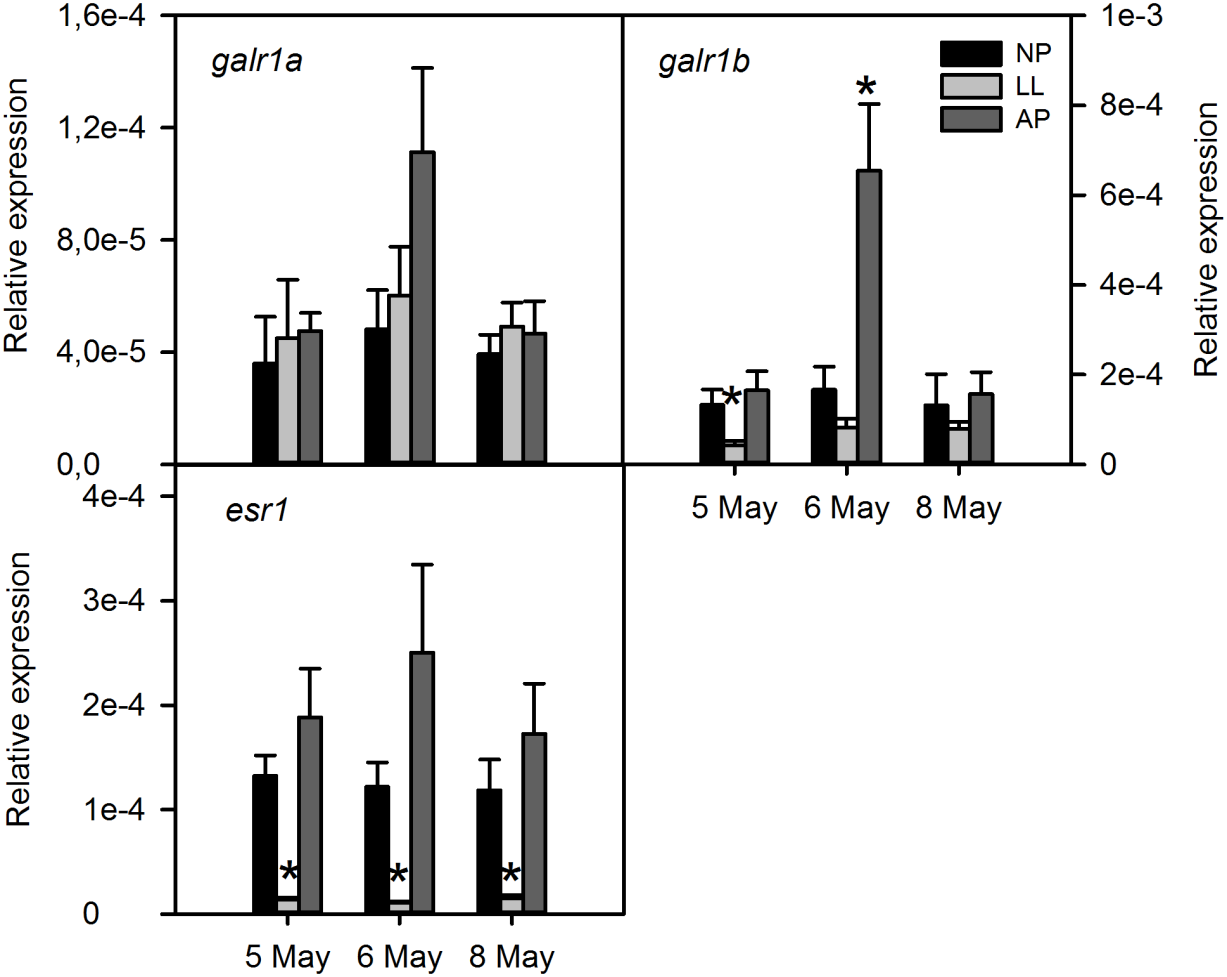
Transcript levels of galanin 1 and estrogen 1 receptors in the brain of sea bass exposed to different photoperiods. The transcript levels of *galr1a*, *galr1b* and *esr1* were measured in whole brains of sea bass exposed to normal (NP), accelerating (AP) and continuous light (LL) using qPCR. Each value is the mean ± SEM (n = 6 fish per group at each sampling time) of the relative expression. Asterisk (*) indicates significant differences of AP and LL fish relative to control fish (NP) (P<0.05).

The *npas2* mRNA levels were up regulated in the LL group compared to the NP control (Fig 3). In contrast, *gnrh1*, *gnrh2*, *kiss2* and *esr1* were significantly down regulated compared to NP (Figs 4 and 5). The *galr1b* mRNA levels were globally down regulated in the LL group compared to the NP control although there was only statistical significance in the second time point (day 2) analysed (Fig 5). The *kiss1rb* was up regulated in the AP group at all-time points and in the LL at the second time point (Fig 4). The *clock* and *galr1a* gene expression in the LL group did not differ from the NP group (Figs 3 and 5, respectively).

The *gnrh1*, *gnrh2*, *kiss1rb* and *npas2* responded with a similar pattern in the AP group with up regulation at day 1 which remained elevated for at least 4 days compared to the NP group (Figs 3 and 4). The *kiss2* and *galr1b* were upregulated only at 48 hours (Figs 4 and 5). The *clock1*, *esr1* and *galr1a* mRNA levels in the AP and the NP group were similar (Figs 3 and 5).

## Discussion

In the present study we have identified a set of co-regulated candidate genes potentially involved in photoperiod induced onset of puberty in fishes. We also observed modulation of transcription of key puberty related genes in the brain of immature sea bass by inhibiting (LL) or accelerating (AP) photoperiods and this is likely to represent an early response to photoperiod before it becomes detectable at the endocrine or morphological level.

### Co-regulation of circadian clock genes and the *gnrh2-kiss-kissr* system in fish reproduction

Interestingly, *kiss1*, *kiss2*, *kissr* and *gnrh2* gene promoters shared the same TF frameworks with the master key clock genes *clock*, *npas2* and *arntl1*. Moreover, additional genes with the same promoter modules were identified allowing us to predict gene networks that may be co-regulated with the circadian and *kiss*/*kissr*-*gnrh2* system. When the *gnrh2* promoter was included in the analysis the list of co-regulated genes overlapped only 3% compared to the list obtained in its absence, clearly demonstrating the specificity of the promoter modules and of the predicted gene network partners. In addition, only when *gnrh2* was included in the analysis with *clock*, *npas2*, *arntl1*, *gnrh2* and *kiss*/*kissr* was reproductive function predicted as the main biological process. These results not only support the hypothesis that circadian clock genes *clock*, *npas2*, *arntl1*, and the *kiss*/*kissr*-*gnrh2* system are co-regulated but also suggest a putative role for the still enigmatic function of *gnrh2* in reproduction.

In sea bass the main reproductive events are notably sensitive to environmental factors and responsiveness appears to be determined by size, with larger males maturing earlier. Such observations led to suggestions that metabolic/nutritional cues may be involved in the activation of pubertal development in precocious males [17,31]. However, to date none of the factors that stimulate (orexigenic, e.g. galanin, growth hormone, somatostatins) or inhibit (anorexigenic, e.g., leptin, agouti, glucagon) food intake have been linked to the activation of puberty in fish [32–34]. The *in silico* analysis suggests a common regulation of the circadian clock and reproduction, as well as appetite and growth regulating, genes e.g., galanin receptor (*galr1*), glucagon receptor 1 (*gcgr-1*), growth hormone receptor (*ghr*), leptin, agouti signalling peptide (*asip*) and somatostatin (*sst*). Interestingly, recent experimental evidence implicates also Gnrh2 in the suppression of appetite in zebrafish [35] and *kiss2r* has been identified in somatostatin positive neurons in sea bass [36].

In sea bass, 4 galanin receptors have been described: *galr1*a/b and *galr*2a/b, of which *galr1a* and *galr1b* are highly stimulated in the testes by 11-KT treatments [30]. Interestingly, we found that brain *galr1b* mRNA levels showed signs of suppression by LL. These results together with this receptor’s androgen sensitivity in the testes of pre-pubertal fish [30] suggests a role in reproduction for the galinergic system acting in the brain and in peripheral tissues (i.e. testes).

### Differential regulation of circadian clock components (*clock* and *npas2*) by light

The mechanisms by which photoperiod entrains the rhythmicity of reproductive processes and translates them into hormonal cues are still poorly understood. The *ARNTL1* and *CLOCK*/*NPAS2* genes are good candidates for this role as their interaction and dimerization drives the rhythmicity of downstream clock gene, i.e. *PER1*, *PER2*, cryptochrome circadian clock 1 and 2 (*CRY1* and *CRY2*, respectively) expression/translation [8]. In agreement with this view is the identification of common regulatory TFBS frameworks in *clock*, *npas2*, *arntl1*, *per2*, prokineticin2 (*pk2*) and nuclear factor interleukin 3 regulated (*e4bp4*/*nfil3*) gene promoters, suggesting that they are co-regulated by the same factors. In addition, the likelihood of co-regulation with *gnrh2*, *kiss/kissr* further supports their involvement in fish reproduction. However, we found that the expression of *clock* was not influenced by LL or AP treatments. Conversely, the transcription of *npas2* was highly inducible by light treatments as both photoperiod regimes upregulated the transcription of this gene. The lack of effect of light on *clock* expression suggests that the transcription of this gene is probably not directly modulated by light and that the early responses to light most likely rely on *npas2* as an upstream regulator of the circadian clock in the brain. Nevertheless, the opposing effects of LL and AP on pubertal development in sea bass cannot be explained by a specific effect of light regime on the transcription of these key master clock genes during the period analysed.

### The *kiss2*, *gnrh1* and *esr1* gene expression is down regulated by LL

The prediction by framework analysis of co-regulation of *kiss1*/*kiss2* paralogs and their receptors *kiss1rb*/*kiss2rb* with circadian clock genes and *gnrh1* and *gnrh2* supports the hypothesis that the kiss/kissr system is likely to be involved in the translation of the light stimulus to activate the reproductive axis in the sea bass. Interestingly, LL fish not only had suppressed brain *kiss2* but also suppressed *gnrh1* levels, suggesting that the regulation of these genes is not independent. These results are also in agreement with previous work demonstrating that kiss 2–12 intracerebroventricular injections upregulate *gnrh1* gene expression (and peptide release) and induce a surge in plasma Lh in pre-pubertal sea bass [37]. In pre-pubertal sea bass, long photoperiods disrupt and continuous light completely abolishes melatonin circadian rhythms, and in the latter case Lh circadian rhythm in the pituitary is abolished without affecting the rhythms of Gnrh1 release [38,39]. This suggests that the circadian Lh levels are entrained by a light-dependent mechanism and that a Gnrh1 upstream effector may be involved in this regulation. Based on the above, kisspeptins are the likely candidates for this role which is supported by the observation of projections of kiss secretory neurons to the pituitary in striped bass and zebrafish [40]. Taken together these observations may indicate that the continuous light-induced delay in pubertal development in sea bass may “damp” *kiss2 and gnrh1* signals so that they fail to activate gonadotropin pulses and steroid production at puberty onset. Whether this is a direct effect of light on gene expression or somehow related to disrupted melatonin levels is still an open question.

Although we were not able to measure significant levels of brain *kiss1* transcripts in the present study, we have analysed the effect of photoperiod on the transcription of the putative *kiss*1 receptor, *kiss1rb* [22]. There were no differences between fish exposed to LL and NP on *kiss1rb* expression. However, we found a significant suppressive effect of the LL photoperiod on brain estrogen receptor 1 (*esr1*) mRNA levels, which has recently been shown to be co-expressed with *kiss1* in the medio-basal hypothalamus cells [41], opening a possibility for a possible role of *kiss1* in this process. Nevertheless, further work is needed to clarify this possibility.

### *kiss2*, *kiss1rb*, *gnrh1* and *gnrh2* gene expression are up regulated by accelerating photoperiod regimes

We further analysed the gene expression of *kiss2*, *kiss1rb*, *gnrh1* and *gnrh2* in fish exposed to a shift from a long onto a short photoperiod, which provides for an effective accelerating signal to trigger the onset of puberty. Interestingly we have found that the *kiss2* mRNA levels were not so markedly affected in AP compared to LL fish. The same pattern was also found for *galr1b*, as both genes were only affected 48 h after the light shift. Although we cannot fully explain the significance of these results, it is possible that either this increase reflects a re-phasing of gene expression in response to the photoperiod shift or that the rapid and transient increase in these genes may be part of the early events of the photo-stimulation of the BGP axis. Conversely, both *gnrh 1* and *2* and *kiss1rb* were significantly upregulated throughout the 4 day period following the shift in photoperiod, which could indicate that the positive effect of AP regimes on sea bass puberty may be linked to a stimulatory effect on the *gnrh1/kiss1/kiss1rb* system in the brain. Nevertheless, more work is necessary to show whether *kiss1* levels are also affected by photoperiod.

Interestingly, *gnrh1* expression appears to be more responsive to the decrease in photoperiod than to the absence of a dark phase. While sea bass pituitary Gnrh1 levels oscillate with the circadian light/dark cycle [38], Gnrh2 appears to be involved in the amplification of nocturnal melatonin release from the pineal gland [42]. This role in melatonin regulation may explain why in the present study *gnrh2* was strongly induced when the dark phase was increased (AP) but not when it was eliminated (LL). Whether the photoperiodic modulation of *gnrh2* levels in the brain may impact the reproductive function is still not clear. In sea bass, *gnrh2* expressing cells are detected near the pituitary stalk but no neuronal projections to the pituitary have been detected to date [43]. However, it is possible that *gnrh2* neuron projections may exist when fish are exposed to certain environmental and/or physiological challenges as demonstrated in zebrafish [44].

Conversely, unlike in LL, *esr1* mRNA levels were not significantly affected in fish exposed to AP. The lack of effect of stimulatory light regimes in opposition to the strongly suppressive effect of inhibitory light regimes may indicate that estrogen metabolism does not play a central role in the activation of puberty, but may have agonistic effects on other key signalling pathways.

## Conclusions

**Fig 6:**
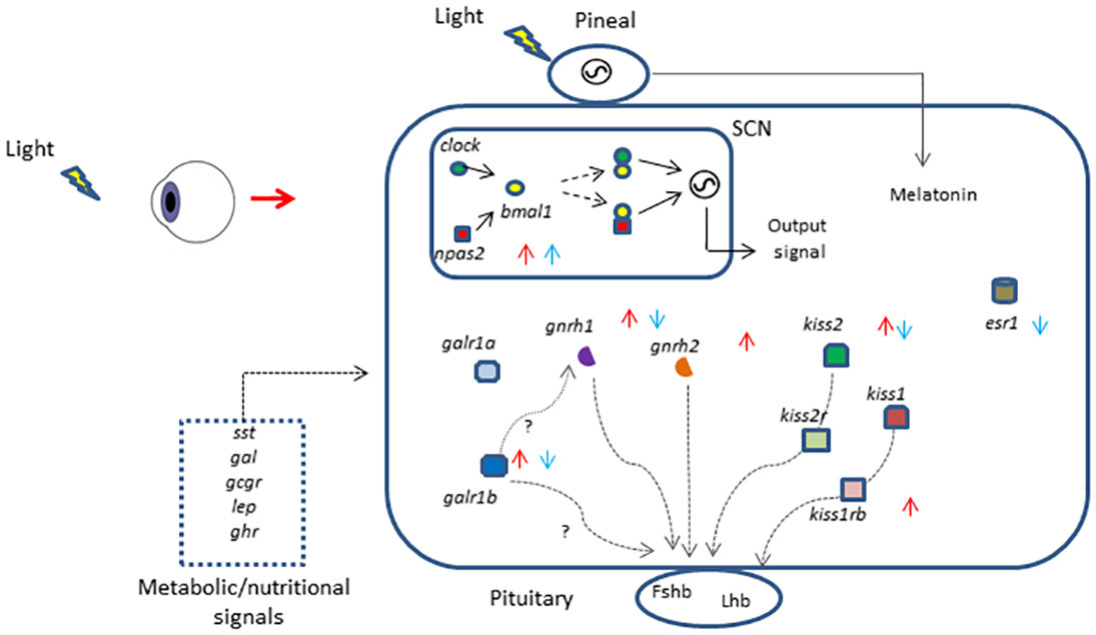
Diagram summarizing the results obtained in this study. The key genes involved in gonadotropin stimulation and activation of the BPG axis are highly responsive soon after photoperiodic shift and prior to pubertal activation. Accelerating photoperiod regimes, AP (red arrows), increased *npas2*, *gnrh1*, *gnrh2*, *kiss2* and *kiss1rb*, demonstrating that light not only affects the circadian clock but also key genes involved in gonadotropin stimulation. Continuous light, LL (blue arrows), down-regulated *gnrh1*, *kiss2* and *esr1* but had no effect on *kiss1rb*. Several candidate genes involved in signalling the metabolic/nutritional status of the individual were identified and *galr1b* gene expression was upregulated by AP and down-regulated by LL. *sst*-somatostatin, *gal*- galanin, *gcgr*- glucagon receptor, *lep*- leptin, *ghr*- growth hormone receptor, *clock*- circadian locomotor output cycles kaput, *npas2*-neuronal PAS domain protein 2, *bmal1* (*artnl*)- Aryl hydrocarbon receptor nuclear translocator-like, *galr1a*- galanin receptor 1a, *galr1b*- galanin receptor b, *esr1*- estrogen receptor 1, *gnrh1*- gonadotropin releasing hormone 1, *gnrh2*- gonadotropin releasing hormone 2, *kiss1*- kisspeptin 1, *kiss2*- kisspeptin 2, *kiss1rb*- kisspeptin 1 receptor b, *kiss2r*- kisspeptin 2 receptor, FSHb-follicle stimulating hormone beta, LHb- luteinizing hormone beta, SCN-suprachiasmatic nucleus.

Altogether, the results presented herein show that the transcription of *npas2*, *gnrh1*, *gnrh2*, *kiss2*, *kiss1rb*, *galr1b* and *esr1* is responsive to stimulating and inhibiting photoperiods in pre-pubertal sea bass. Circadian clock genes in the brain in response to photoperiod alterations, possibly through *npas2/bmal1* stimulation (Fig 6), may play a role in the signalling cascade involved in triggering puberty. LL appears to affect *gnrh1*, *kiss2* and estrogen signalling (via *esr1*), while AP appears to affect *gnrh2*, *gnrh1* and *kiss1rb* (Fig 6). Gene promoter analysis predicted nutritional/metabolism related genes within the reproduction related gene network, and one such gene, *galr1b*, was highly responsive to photoperiod treatments (Fig 6). Regardless of the photoperiod regime, *gnrh1* was markedly affected by LL and AP suggesting a central role in the integration/translation of light stimuli to the reproduction-related gene network associated with pubertal onset.

## Supporting Information

S1 Fig Size grading at the start of the photoperiod experiment. Fish were separated as larger (class 1) as or smaller than 3g (class 2). Boxes represent the 35 and 75 percentiles, whiskers the 10 and 90 percentiles and the mid line is the median.
(TIF)

S1 Table TF frameworks identified in zebrafish gene promoters. (DOCX)

S2 Table TF frameworks identified in zebrafish gene promoters. (DOCX)

S3 Table Circadian clock-*gnrh2* and *kiss*/*kissr* gene network partners. (XLSX)

S4 Table Circadian clock-*kiss*/*kissr* gene network partners. (XLSX)

S5 Table Clustering analysis of circadian clock-*gnrh2*-*kiss*/*kissr* gene network partners. (XLSX)

S6 Table Clustering analysis of circadian clock-*kiss*/*kissr* gene network partners. (XLSX)
