# Supplementary material for: Photoperiodic Modulation of Circadian Clock and Reproductive Axis Gene Expression in the Pre-Pubertal European Sea Bass Brain
Source: PLoS One. 2015 Dec 7;10(12):e0144158. doi: 10.1371/journal.pone.0144158 (PMC4671726; doi:10.1371/journal.pone.0144158)
Supplement: S1 Table — (DOCX) [file pone.0144158.s002.docx]

**Supplementary file 2**- Common transcription factor frameworks identified in zebrafish circadian clock and Kiss/Gpr54s-GnRH genes.

|  | Transcription factor binding sites and distances | | | | | |
| --- | --- | --- | --- | --- | --- | --- |
| Frameworks^(a)^ | Element 1^(b)^ | Distance (bp) ^(c)^ | Element 2^(b)^ | Distance (bp) ^(c)^ | Element 3^(b)^ | p-value^(d)^ |
| 1 | V$HOMF | 11-28 | V$OCT1 | 54-72 | V$BRNF | 5,60E-17 |
| 2 | V$SORY | 243-259 | V$BRNF | 7-25 | V$BRNF | 2,00E-15 |
| 3 | V$FKHD | 5-19 | V$CREB | 11-30 | V$BRNF | 7,68E-15 |
| 4 | V$BRNF | 7-20 | V$BRNF | 214-227 | V$BRNF | 8,60E-15 |
| 5 | V$FKHD | 24-43 | V$BRNF | 7-25 | V$CART | 1,50E-14 |
| 6 | V$BRNF | 7-19 | V$BRNF | 339-354 | V$HOXF | 4,01E-13 |
| 7 | V$BRNF | 105-117 | V$HOXF | 5-23 | V$BRNF | 1,66E-12 |
| 8 | V$HOXF | 22-37 | V$BRNF | 62-78 | V$BRNF | 7,13E-12 |
| 9 | V$BRNF | 102-119 | V$HOXF | 12-30 | V$HOXF | 1,20E-11 |
| 10 | V$HOXF | 20-37 | V$BRNF | 5-18 | V$HOXF | 2,38E-11 |

^(a)^ Conserved frameworks models identified in circadian clock and KISS/GPRs-GnRHs promoter genes;

^(b)^ Specific transcription factor matrixes identified in each framework model: V$HOXF- Paralog hox genes 1-8 from the four hox clusters A, B, C, D; V$BRNF- Brn POU domain factors; V$CART- Cartilage homeoprotein 1; V$FKHD- Forkhead domain factors; V$CREB- cAMP responsive element binding proteins; V$SORY- SOX/SRY-sex /testis determining and related HMG box factors; V$HOMF- Homeodomain transcription factors and V$OCT1- Octamer binding protein;

^(c)^ Distance in base pairs (bp) between transcription factors within each framework;

^(d)^ Each of the framework models identified was compared to a background promoter sequence set of 5000 human promoters and a p-value was attributed to denote its specificity.
