# Supplementary material for: Photoperiodic Modulation of Circadian Clock and Reproductive Axis Gene Expression in the Pre-Pubertal European Sea Bass Brain
Source: PLoS One. 2015 Dec 7;10(12):e0144158. doi: 10.1371/journal.pone.0144158 (PMC4671726; doi:10.1371/journal.pone.0144158)
Supplement: S2 Table — (DOCX) [file pone.0144158.s003.docx]

**Supplementary file 3**- Common transcription factor frameworks identified in sea bass circadian clock and Kiss/Gpr54s-GnRH genes.

|  | Transcription factor binding sites and distances | | | | |
| --- | --- | --- | --- | --- | --- |
| Frameworks^(a)^ | Element 1^(b)^ | Distance (bp) ^(c)^ | Element 2^(b)^ | Distance (bp) ^(c)^ | Element 3^(b)^ |
| 1 | V$HOMF | 6-39 | V$OCT1 | 20-70 | V$BRNF |
| 2 | V$SORY | 186-268 | V$BRNF | 4-35 | V$BRNF |
| 3 | V$FKHD | 4-29 | V$CREB | 6-46 | V$BRNF |
| 4 | V$BRNF | 4-37 | V$BRNF | 207-280 | V$BRNF |
| 5 | V$FKHD | 7-28 | V$BRNF | 8-30 | V$CART |
| 6 | V$BRNF | 4-28 | V$BRNF | 284-345 | V$HOXF |
| 7 | V$BRNF | 91-129 | V$HOXF | 3-35 | V$BRNF |
| 8 | V$HOXF | 10-33 | V$BRNF | 22-85 | V$BRNF |
| 9 | V$BRNF | 25-114 | V$HOXF | 13-58 | V$HOXF |
| 10 | V$HOXF | 20-110 | V$BRNF | 8-26 | V$HOXF |

^(a)^ Conserved frameworks models identified in sea bass circadian clock and KISS/GPRs-GnRH2 promoter genes;

^(b)^ Specific transcription factor matrixes identified in each framework model: V$HOXF- Paralog hox genes 1-8 from the four hox clusters A, B, C, D; V$BRNF- Brn POU domain factors; V$CART- Cartilage homeoprotein 1; V$FKHD- Forkhead domain factors; V$CREB- cAMP responsive element binding proteins; V$SORY- SOX/SRY-sex /testis determining and related HMG box factors; V$HOMF- Homeodomain transcription factors and V$OCT1- Octamer binding protein;

^(c)^ Distance in base pairs (bp) between transcription factors within each framework.
